# Female social and sexual interest across the menstrual cycle: the roles of pain, sleep and hormones

**DOI:** 10.1186/1472-6874-10-19

**Published:** 2010-05-27

**Authors:** Chrisalbeth J Guillermo, Heidi A Manlove, Peter B Gray, David T Zava, Chandler R Marrs

**Affiliations:** 1 School of Community Health Sciences, University of Nevada, Las Vegas; Las Vegas, NV, USA; 2 Department of Anthropology, University of Nevada, Las Vegas; Las Vegas, NV, USA; 3 ZRT Laboratory; Beaverton, OR, USA; 4 Department of Psychology, University of Nevada, Las Vegas; Las Vegas, NV, USA

## Abstract

**Background:**

Although research suggests that socio-sexual behavior changes in conjunction with the menstrual cycle, several potential factors are rarely taken into consideration. We investigated the role of changing hormone concentrations on self-reported physical discomfort, sleep, exercise and socio-sexual interest in young, healthy women.

**Methods:**

Salivary hormones (dehydroepiandrosterone sulfate-DHEAS, progesterone, cortisol, testosterone, estradiol and estriol) and socio-sexual variables were measured in 20 women taking oral contraceptives (OC group) and 20 not using OCs (control group). Outcome measures were adapted from questionnaires of menstrual cycle-related symptoms, physical activity, and interpersonal relations. Testing occurred during menstruation (T1), mid-cycle (T2), and during the luteal phase (T3). Changes in behavior were assessed across time points and between groups. Additionally, correlations between hormones and socio-behavioral characteristics were determined.

**Results:**

Physical discomfort and sleep disturbances peaked at T1 for both groups. Exercise levels and overall socio-sexual interest did not change across the menstrual cycle for both groups combined. However, slight mid-cycle increases in general and physical attraction were noted among the control group, whereas the OC group experienced significantly greater socio-sexual interest across all phases compared to the control group. Associations with hormones differed by group and cycle phase. The estrogens were correlated with socio-sexual and physical variables at T1 and T3 in the control group; whereas progesterone, cortisol, and DHEAS were more closely associated with these variables in the OC group across test times. The direction of influence further varies by behavior, group, and time point. Among naturally cycling women, higher concentrations of estradiol and estriol are associated with lower attraction scores at T1 but higher scores at T3. Among OC users, DHEAS and progesterone exhibit opposing relationships with attraction scores at T1 and invert at T3.

**Conclusions:**

Data from this study show no change across the cycle in socio-sexual interest among healthy, reproductive age women but higher social and physical attraction among OC users. Furthermore, a broader range of hormones may be associated with attraction than previously thought. Such relationships differ by use of oral contraceptives, and may either reflect endogenous hormone modulation by OCs and/or self-selection of sexually active women to practice contraceptive techniques.

## Background

A growing body of research addresses the psychological and behavioral changes women experience across the menstrual cycle. Much of that research focuses on mood and sexuality [e.g. [1]]. Subtle shifts in female libido across the menstrual cycle and somewhat less noticeable changes in socio-sexual behavior have also been noted [2,3]. However, comparisons between these types of effects among normally cycling women and women using oral contraceptives (OCs) have not been fully investigated. Moreover, the underlying associations between cyclical and OC-mediated hormone changes and socio-sexual behavior are not clearly understood.

In the present paper, we consider the possible role of changing hormone concentrations on fluctuations in women's self-reported physical discomfort, sleep, exercise and socio-sexual interest from the functional perspective. According to this perspective, behavioral changes that support favorable mating strategies coincide with times of high fertility, ovulation, and higher estrogen concentrations [4]. Conversely, behavioral changes that promote pregnancy such as decreased sexual and physical activity and increased sleep are likely to occur during the luteal or high progesterone phase of the woman's menstrual cycle. Finally, the observed sleep, activity and mood disruptions that often develop pre- and peri-menstrually, in the absence of conception, may be byproducts of declining hormone levels [5].

A number of projects have supported a functional role for cyclic socio-sexual behavioral changes [6,7]. Around the time of ovulation, normally cycling women commonly experience increased libido, sexual interest and attraction towards potential mates [8]. Women report feeling more attractive themselves, have more interest in attending events where they might meet men, experience more mate guarding by their partner [9] and are more likely to provide a phone number to a prospective mate during the late follicular/high estrogen phase [10]. Conversely, during the luteal phase or high progesterone phase, women report higher commitment to their relationships [11]. They also tend to prefer more feminized male faces which has been postulated to represent a shift towards a desire for longer term social support [11].

Interestingly, researchers have observed similar relationships with other hormones. Specifically, female sexual function has been shown to increase during the ovulatory phase of the menstrual cycle, in congruence with rising free and total testosterone [12]. This is in opposition to the work of Battaglia et al. [13], who report no significant variation in androgens across the cycle but mention that additional synthesis and metabolism at the adrenal glands and peripheral tissues make it difficult to accurately correlate a specific androgen to sexual function.

Together, these data suggest women's socio-sexual interests and behaviors fluctuate across the menstrual cycle, in concert with changes in fertility and circulating hormone concentrations. How the use of OCs affects these variable is not clear. Although, OCs have been reported to attenuate the cyclicity of oxytocin, a pituitary hormone known to increase during sexual arousal and orgasm, whereas the influence of oxytocin on lubrication is maintained despite lacking cyclic variation [14]. Researchers have been otherwise unable to identify consistent patterns in socio-sexual behavior among women using OCs [10,15-17]. This may reflect the lower concentrations and smaller variations in endogenous hormone concentrations found in women using OCs or reactions mediated by the synthetic hormones themselves.

Our aims in the present study are to: 1) examine whether women experience alterations in physical discomfort, sleep quality, exercise, and socio-sexual interest across the cycle; 2) evaluate whether normally cycling women experience different patterns in these outcomes compared with women on OCs; and 3) identify the hormonal correlates of these outcomes at different stages in the cycle, both in normally cycling women and women using OCs.

## Methods

### Participants

This research is part of a larger study to gain insight into the psychological, social, and behavioral aspects of steroid hormones and the menstrual cycle. A full description of other study measures is provided in Guillermo et al. (in review). Healthy, non-smoking, and regularly cycling women between the ages of 18-30 were recruited from the student population at the University of Nevada, Las Vegas. This study was approved by the University of Nevada, Las Vegas Institutional Review Board and all participants voluntarily provided a written, informed consent prior to enrollment.

Individuals were excluded if they reported diagnosis of mental illness, endocrine disease, substance abuse, medication use or medical conditions that would alter hormone concentrations; this includes the use of OCs among the control group within the last six months of enrollment. Additionally, women were excluded from analysis if they had irregular cycles, as classified by having less than 25 or greater than 40 days and deviating more than 10 days from self-reported average cycle length. Fifty-six women enrolled in the study; however, nine were lost to attrition and seven were excluded due to irregular menstrual cycles. As a result, twenty normally cycling women were assigned to the control group and twenty women using oral contraceptives were assigned to the OC group.

Physical discomfort, sleep disturbance, exercise levels, socio-sexual interests, and hormone values were assessed three times during the course of a single menstrual cycle: early- to mid-follicular phase (T1), mid-cycle (T2), and the mid- to late-luteal phase (T3) corresponding to roughly days 7, 14, and 21 for a 28-day cycle. Specimen collection days and subsequent test times were determined based on individually reported average menstrual cycle length and adjusted for the start date of the next menses after the enrollment interview.

### Outcome Measures

At each session, a questionnaire was administered to measure change in behavior across the menstrual cycle. The questionnaire was derived from sections of several social behavior and attraction questionnaires that included questions regarding physical activity (International Physical Activity Questionnaire; "IPAQ", 2002), sleep patterns (Karolinska Sleep Questionnaire) [17,18], and interpersonal relations (Interpersonal Attraction Scales) [19,20]. Questions regarding menstrual cycle related physical discomfort (cramping, bloating, breast tenderness, etc.) were derived from Premenstrual Symptom Daily Rating Scale [21,22]. A question of general physical activity was included, in which the women were asked about the average amount of vigorous physical activity (minutes/hours per day) over the last three days. Questions rating general attraction, as well as social, physical/sexual, and task-oriented attraction, towards a current or potential significant other were also asked. All questions utilized a 5-point Likert-type scale (i.e. 0 = "not at all" to 4 = extremely). For the purposes of data analyses, questions were categorized by content and analyzed by category. Individual questions and categories are listed in Table 1.

**Table 1:** Description and list of individual questionnaire items for each socio-behavioral category.

Behavior
Physical Discomfort (0 = lowest frequency, duration, severity; 4 = highest frequency, duration, severity)
Cramps and/or abdominal pain
Bloating and/or weight gain
Breast tenderness and swelling

Exercise (hours per day)
Participate in vigorous physical activity as part of work, school, home, or leisure time

Sleep Disturbance (0 = statement does not apply at all; 4 = statement always applies)
Experience repeated awakenings
Feel exhausted upon waking
Do not feel well-rested after sleeping
Fight falling asleep during the day
Fall asleep at work, school, or activities

General Attraction (0 = not at all; 4 = extremely)
Feel an attraction or interest toward another person

Social Attraction
Would be difficult to approach other (reverse-scaled)
Would not be able to establish a friendly rapport with other (reverse-scaled)
Other would understand how I feel
Would enjoy the company of other

Physical Attraction
Would find other physically attractive
Other would be sexually appealing
Would take pleasure in sexual relations with other

Task-oriented Attraction
Would not get anything accomplished with other (reverse-scaled)
Would be able to depend on other to get things done
Other would be fun to work with

### Hormone collection procedures

Pre-prandial, morning salivary specimens were collected via expectoration prior to arrival at the testing facility. Hormone specimens were stored in a -25°C freezer until batch analyses by a commercial, CLIA certified laboratory (ZRT Laboratory; Beaverton, OR) using indirect enzyme-linked immunosorbent assay (ELISA) techniques. All hormone testing supplies and analyses were provided and conducted by ZRT Laboratory. Inter- and intra assay coefficients of variation did not exceed 15% for any analyte.

### Statistical Analysis

Category scores were based upon the summed scores of individual questions within each category. Attraction questions that reflected a negative interaction with a current or potential significant other were reverse-scaled to maintain consistency in analysis. Within-cycle and between-group differences were calculated using a mixed-model analysis of variance (ANOVA). Assumptions of sphericity for each variable were evaluated using Mauchly's W statistic. If sphericity was violated, adjustments to the significance levels were made using the Greenhouse-Geisser method. All analyses were calculated using the statistical software SPSS 16.0 and PASW 17.0 (formerly SPSS).

Between-group comparisons of demographic features were assessed through Student's t-test. Between-group comparisons of physical discomfort, sleep, exercise and socio-sexual categories were calculated using a series of one-way ANOVAs for each category and by time point. Associations between hormones and outcome variables were calculated using Pearson Product Moment Correlations. Normality was assessed and non-normally distributed values were log10-transformed prior to analysis to meet the normality assumptions of the test. Only testosterone was normally distributed and did not require transformation. Correlations for estriol, which could not be normalized by transformation, were calculated using Spearman's Rho. Post-hoc correlations between individual questions and hormone concentrations were calculated to evaluate more fully the trends in socio-sexual interest.

## Results

### Participant characteristics

Table 2 summarizes the demographic data by group. Mean test days were T1 = 5.78 (SD 2.09), T2 = 14.25 (SD 1.77), T3 = 22.50 (SD 2.34) for all groups combined. No differences were found between age, education or body mass index (BMI), whereas a higher proportion of Asian/Pacific Islanders were in the control group in comparison to the OC group. Although the distribution of BMI was not statistically significant, more overweight (25 < BMI < 30) women were naturally cycling (control n = 5; OC n = 2) and more obese (BMI > 30) women were OC users (control n = 1; OC n = 4).

**Table 2:** Descriptive statistics of demographic data by group (frequency and proportion or mean and SD, as appropriate).

Characteristic		Control Group	OC Group	T-Test
Age		20.3 (2.56)	20.5 (1.79)	n.s.
Education (yrs)		14.8 (1.68)	15.4 (1.93)	n.s.
BMI		23.3 (3.85)	24.3 (4.50)	n.s.
Cycle length		30.5 (4.12)	27.6 (1.29)	t = 2.99, p < 0.01
Test Day				
Follicular		5.6 (2.24)	6.0 (1.97)	n.s.
Mid-Cycle		14.5 (2.21)	14.0 (1.17)	n.s.
Luteal		22.8 (3.00)	22.2 (1.44)	n.s.
Race/Ethnicity				
Caucasian/NHW		8 (40%)	11 (55%)	
African American/Black		1 (5%)	1 (5%)	
Hispanic/Latino		5 (25%)	5 (25%)	
Asian/Pacific Islander		6 (30%)	3 (15%)	

Education based on years from kindergarten through college; BMI: Body Mass Index; NHW: Non-Hispanic White.

Hormone preparations varied greatly, thus preventing any post-hoc analysis with sufficient statistical power. Of the twenty OC users' pill regimens, 8 were low-estrogen (20-25 μg) versus high-estrogen (30-35 μg) and 5 were multiphasic versus monophasic. Among the different progestin components, 5 women were using OCs containing drospirenone, 5 norgestimate, 4 levonorgestrel, 4 norethindrone, 1 desogestrel, and 1 norgestrel.

### Alterations in physical discomfort, exercise, sleep disturbance, and socio-sexual interest across the cycle

As illustrated in Table 3, participants experienced a significantly greater physical discomfort and more disturbed sleep during the follicular phase as compared to any other phase of the menstrual cycle. No cycle-related changes were observed in exercise, or the general, social, and task attraction scales.

**Table 3:** Comparison of socio-behavioral categories (max possible scores) by phase in menstrual cycle.

Behavior	Statistic	Follicular	Mid-Cycle	Luteal	Main Effect	T1 vs. T2	T2 vs. T3
Physical Discomfort (36)	Mean	13.83	2.55	3.45	46.19***	68.09***	0.79
	SD	8.81	5.35	6.99			
	Range	0-33	0-27	0-33			
Exercise (hr/day)	Mean	1.21	0.74	1.09	1.94		
	SD	1.94	0.75	1.47			
	Range	0-8	0-2	0-5			
Sleep Disturbance (20)	Mean	8.40	4.90	4.68	25.22***	38.82***	0.24
	SD	4.06	3.83	4.26			
	Range	1-18	0-16	0-19			
General Attraction (4)	Mean	2.05	2.28	2.00	1.12		
	SD	1.43	1.20	1.16			
	Range	0-4	0-4	0-4			
Social Attraction (16)	Mean	12.28	12.33	12.65	0.41		
	SD	3.05	2.95	2.46			
	Range	5-16	2-16	7-16			
Physical Attraction (12)	Mean	8.45	8.73	8.05	0.96		
	SD	3.29	3.48	3.89			
	Range	2-12	0-12	0-12			
Task-oriented Attraction (12)	Mean	7.90	8.00	8.00	0.05		
	SD	2.32	2.72	2.66			
	Range	2-12	1-12	1-12			

*p < .05, **p < .01, ***p < .001.

T1: early- to mid-follicular phase, T2: mid-cycle, T3: mid- to late-luteal phase.

### Between-group differences in physical discomfort, exercise, sleep disturbance, and socio-sexual behavior

Results of between-group analyses are presented in Table 4. No between-group differences in physical discomfort, exercise or sleep were observed. At each phase of the menstrual cycle, women taking an OC reported significantly higher general attraction toward current or potential mates, significantly higher social attraction across all phases and higher physical attraction, particularly during the follicular phase, compared to the control group. No differences were found in task attraction.

**Table 4:** Socio-behavioral categories (max possible scores) by group and between-group comparisons.

Behavior	Time	Control Group			OC Group			Between-Group	
				
		Mean	SD	Range	Mean	SD	Range	F-value	p-value
Physical Discomfort (36)	Follicular	13.85	10.36	0-33	13.80	7.19	3-29	0.08	0.78
	Mid-Cycle	2.35	6.34	0-27	2.75	4.29	0-13		
	Luteal	2.90	7.85	0-33	4.00	6.16	0-20		
Exercise (hr/day)	Follicular	0.83	1.23	0-4	1.60	2.43	0-8	2.09	0.16
	Mid-Cycle	0.63	0.65	0-2	0.85	0.84	0-2		
	Luteal	0.80	1.11	0-4	1.38	1.74	0-5		
Sleep Disturbance (20)	Follicular	9.20	4.56	1-18	7.60	3.42	2-13	2.58	0.12
	Mid-Cycle	5.60	4.39	0-16	4.20	3.12	0-13		
	Luteal	5.75	5.07	0-19	3.60	3.02	0-9		
General Attraction (4)	Follicular	1.50	1.24	0-4	2.60	1.43	0-4	18.15	<0.001
	Mid-Cycle	1.78	1.15	0-4	2.79	1.06	0-4		
	Luteal	1.31	0.96	0-3	2.68	0.92	1-4		
Social Attraction (16)	Follicular	11.15	3.28	5-16	13.40	2.39	9-16	7.92	<0.01
	Mid-Cycle	11.25	3.49	2-16	13.40	1.79	8-16		
	Luteal	12.00	2.43	8-16	13.30	2.36	7-16		
Physical Attraction (12)	Follicular	6.95	3.44	2-12	9.95	2.37	4-12	5.83	0.02
	Mid-Cycle	7.95	3.79	1-12	9.50	3.03	0-12		
	Luteal	7.00	4.36	0-12	9.10	3.11	0-12		
Task-oriented Attraction (12)	Follicular	7.50	2.37	2-10	8.30	2.25	3-12	1.83	0.18
	Mid-Cycle	7.35	2.92	1-12	8.65	2.39	4-12		
	Luteal	7.65	2.92	1-11	8.35	2.68	3-12		

### Hormonal correlations with physical discomfort, exercise, sleep disturbance, and socio-sexual perceptions

As illustrated by Table 5, relationships between individual hormones and the variables measured differed by group and by cycle phase. For the control group, higher estradiol was associated with more physical discomfort at T1 and more perceptions of physical attraction at T3. Higher estriol, however, was associated with less general attraction at T3. None of the other hormones measured were associated with any other variables.

**Table 5:** Correlations of hormones to socio-behavioral categories and individual questionnaire items.

**Control Group**				**OC Group**			
	
T1	Physical Discomfort	E2	.500*	T1	General Attraction	D	.450*
	Cramp Frequency	E2	.453*		Physical Attraction	E3	-.488*
	Bloating Severity	E2	.658**		Physically Attracted	E3	-.544*
	Bloating Frequency	E3	.447*		Physically Attracted	T	-.543*
	Bloating Severity	T	.526*		Task-Oriented Attraction		n.s.
	Exercise	E2	.575**		Depend on Other	P	-.456*
	Sleep Disturbance		n.s.		Depend on Other	T	-.558*
				
	Awakenings	E3	.481*	T2	Physical Discomfort		n.s.
	Social Attraction	T	-.541*		Bloating Duration	E3	.543*
	Approach Other	E2	-.662**		Bloating Severity	E3	.543*
	Establish Rapport	E2	-.456*		Bloating Frequency	D	.455*
	Approach Other	E3	-.497*		Exercise	D	.496*
	Approach Other	T	-.646**		Sleep Disturbance		n.s.
	Establish Rapport	T	-.544*		Fight Falling Asleep	T	-.456*
	Physical Attraction		n.s.		Social Attraction		n.s.
	Physically Attracted	P	.472*		Approach Other	D	.535*
				
T2	Physical Discomfort		n.s.		Task-Oriented Attraction		n.s.
	Bloating Duration	E3	.543*		Accomplish Work with Other	E2	.465*
	Bloating Severity	E3	.543*		Accomplish Work with Other	E3	.452*
	Bloating Frequency	D	.455*		Accomplish Work with Other	D	.623**
				
	Social Attraction		n.s.	T3	Physical Discomfort	C	-.482*
	Enjoy Company	C	.487*		Breast Tenderness Duration	P	-.454*
				
T3	Sleep Disturbance		n.s.		Breast Tenderness Severity	P	-.470*
	Awakenings	E2	.466*		Cramp Duration	C	-.525*
	General Attraction	E3	-.462*		Breast Tenderness Frequency	C	-.483*
	Social Attraction		n.s.		Breast Tenderness Duration	C	-.479*
	Understood by Other	E2	.474*		Exercise	E2	.594**
	Physical Attraction	E2	.446*		Exercise	P	.461*
	Other Sexually Appealing	E2	.486*		Exercise	T	.572**
	Task-oriented Attraction				Exercise	D	.465*
	Accomplish Work with Other	E3	.564**		Social Attraction		n.s.
					Establish Rapport	P	.556*
					Understood by Other	D	-.466*
					Physical Attraction		n.s.
					Physically Attracted	D	-.446*
					Task-Oriented Attraction		n.s.
					Accomplish Work with Other	P	.507*

*p < 0.05, **p < 0.01, n.s. = non-significant for category associations.

T1: early- to mid-follicular phase, T2: mid-cycle, T3: mid- to late-luteal phase;, E2: estradiol, P: progesterone, E3: estriol, T: testosterone, D: dehydroepiandrosterone sulfate, C: cortisol.

This is in contrast to the OC group where a broader range of hormones was associated with a number of individual indices. For this group, DHEAS was positively associated with general attraction during the follicular phase and exercise levels at mid-cycle and the luteal phase. Follicular estriol concentrations were inversely associated with physical attraction. During the luteal phase, higher estradiol, progesterone, testosterone and DHEAS concentrations were associated with more exercise while cortisol was correlated negatively with physical discomfort.

In an effort to fully understand the role of hormones in socio-sexual behavior, correlations between the individual questions and hormones were calculated (Table 5). Due to the number of correlations, these data should be regarded as trends. Nevertheless, they provide important clues regarding the pattern of hormone-to-behavior relationships in normally cycling and OC-using women. As the table illustrates, associations between hormones and socio-sexual questions, with the exception of progesterone, were negative for both groups during the follicular phase. At mid-cycle, higher cortisol was associated with "enjoying the company of the other" for the control group, whereas higher estradiol and DHEAS concentrations were linked positively to task attraction in the OC group. During the luteal phase, higher estradiol and estriol were associated with sexual interest questions in the control group, whereas higher progesterone was associated with more social attraction and better work interactions in the OC group. In addition, higher luteal DHEAS meant lower social and physical attraction for the OC group.

## Discussion

The monthly window of human female fertility is very short. Functionally, natural selection should favor female behaviors that increase the odds of successful mating around the time of ovulation [4] and preserve the pregnancy, post-conception. We expected 1) each of these reproductive phases to be associated with changes in activity levels and socio-sexual interest, 2) these changes would be mediated by fluctuations in endogenous hormones, and 3) to the extent that OCs altered the natural reproductive cycle, we expected behavioral differences between naturally cycling women and those using OCs. Results from this study revealed that physical discomfort, sleep and sexual interest vary by cycle-phase, but do not appear to influence socio-sexual interest and that OC use and endogenous hormones affect socio-sexual interest, but not in the manner proposed by the functional perspective.

As expected, physical discomfort was significantly more prominent during the follicular phase of the cycle. Moreover, these data are consistent with most other studies finding no general patterns between exercise and cycle phase [23]. Interestingly however, there were no differences in discomfort levels between the two groups of women at any cycle phase. During the follicular phase, estradiol concentrations were significantly associated with discomfort in the control group, but no hormone associations were observed for the OC group. However, during the luteal phase, lower cortisol and progesterone concentrations contributed to more discomfort in the OC group.

In addition to increased physical discomfort during the follicular phase, strong cycle- but not group-related sleep differences were observed. During the follicular phase, both groups reported more sleep disturbances compared to the other phases. Although existing research investigating cycle-related shifts in sleep is sparse, some have observed reductions in REM sleep [24] or poorer sleep [23] during the luteal phase and increases in sleep disturbances in the days prior to and during menses. Researchers postulate that higher luteal phase estradiol concentrations precede both increased physical discomfort and disturbed sleep [25]. Despite a lack of association between combined sleep disturbance scores and hormones, we observed positive correlations for individual questions with estradiol and estriol among the control group during T3 and T1, respectively, and a negative correlation with testosterone among the OC group during T2 (Table 5).

Consistent with a functional perspective, physical attraction trended upward at mid-cycle for normally cycling women [26,27]. This was not the case for women in the OC group, where physical attraction levels were consistently higher across the cycle and significantly elevated compared to the control group. The OC group also scored significantly higher on measures of general and social attraction across all phases of the cycle compared to the control group and exhibited a distinctly different hormone profile relative to these variables.

In contrast to the functional perspective, mid-cycle estradiol was not associated with sexual or social interest, but it was associated with these variables during the luteal phase in the control group. Similarly, progesterone, which per the functional perspective was expected to be negatively associated with luteal phase socio-sexual interest and potentially associated with follicular phase sleep disturbances or physical discomfort, was not correlated with any of these variables in normally cycling women. These findings, though inconsistent with the functional perspective, are partially in-line with the physiological and endocrine investigations of female sexual arousal.

Despite methodological differences among studies of female sexual behavior, several consistent trends emerge. Firstly, sexual activity (e.g. intercourse or masturbation), arousal (vaginal constriction and lubrication) and subjective sexual interest should be considered as separate, albeit interacting, variables with potentially different endocrine mediators. When these variables are dissociated, the data somewhat consistently point to a decrease in sexual activity, but not necessarily sexual interest, during the early follicular phase, with a trend towards increased mid-cycle sexual interests [15,26,28] that may or may not correspond to actual changes in sexual activity. Our data support these trends.

Secondly, both androgens and estrogens play an important role in female sexual arousal, motivation, and activity [12,15]. In most cases, increased androgens such as dehydroepiandrosterone (DHEA), dehydroepiandrosterone sulfate (DHEAS), androstenedione and/or testosterone favor sexual arousal [29-32], but this relationship may be age, concentration and cycle-phase dependent. Mid-cycle testosterone tends to increase sexual arousal, whereas during the follicular and luteal phases this trend is reversed [33,34]. In contrast, higher progesterone and estradiol concentrations are associated with lower sexual interest, but may also be, age, concentration and cycle-phase dependent [12,34]. To our knowledge, neither cortisol nor estriol have been investigated in association with female social or sexual interest across the menstrual cycle.

In the present study, women using OCs reported significantly higher social and sexual interest across all test times, with no significant fluctuations related to cycle-phase. The lack of cycle-related changes in sexual interest may be related to suppression or leveling of endogenous hormones by their synthetic counterparts [14,28] or may simply be a matter of self-selection to this particular group, in that women in the OC group were likely more often involved in sexual relationships than women in the control group. Some evidence suggest a small minority of women using OCs report diminished sexual interest [28,35-37], whereas other research shows no change [38] or increased interest and activity [39,40]. The effects of oral contraceptives on sexual arousal and activity are not well understood and likely depend as much upon individual hormonal and socio-sexual parameters as on the type and dosage of the synthetic steroids administered.

Similar to the control group, hormone-to-socio-sexual behavior relationships in the OC group were complex and multi-directional. With DHEAS as the exception, negative associations between hormones and socio-sexual variables were observed both at T1 and at T3, whereas positive associations were observed at mid-cycle. In contrast to the control group, however, DHEAS and progesterone played a more prominent role in socio-sexual interest. The altered influence of progesterone is not unexpected as results showed that women in the OC group had significantly lower progesterone than the control group at both T2 and T3 (Guillermo et al., in review). Moreover, although our study did not show any significant differences in DHEAS between controls and OC users at any time point, others have shown that women who use OCs have lower DHEAS than non-users [41]. Hence, both DHEAS and progesterone may exert more influence at lower levels.

Unfortunately, a larger sample is necessary to resolve such complex interactions in a manner that is both meaningful and valid. Although the sample size of 20 women in each of the cycling and OC groups is modest, it is comparable to those employed in similar types of study designs. Additionally, this study is subject to further limitations. The women were young and healthy and thus, their experiences may not generalize to older populations of women. That said, a younger sample may provide more accurate results among women with normal sexual function.

Study duration was approximately one hour for each test session; thus, no assessments were given to confirm participant mental health status, sexual preference, sexual function and activity, or average cycle length. Although participants were not asked whether they engaged in heterosexual, homosexual, or bisexual relationships, research has shown that individuals in homosexual relationships report higher sexual desire as compared to those in heterosexual relationships [42]. Additionally, the compiled questionnaire has not been previously validated with a sufficient sample. Hence, although the outcome measures appear to have tapped some of the desired outcomes, findings are vulnerable to inherent problems with self-reported data and may not be sufficiently sensitive to detect what could be subtle changes in, not only female socio-sexual interest, but also sexual functioning.

Despite these limitations, data from the present study provide a rare opportunity to evaluate variations in female socio-sexual interest across the menstrual cycle in both naturally cycling women and those who utilize oral contraceptives in correspondence with endogenous hormones, sleep, physical discomfort, and activity levels. These data show a mid-cycle increase in sexual interest for normally cycling women and thus, partially support the functional perspective of female mating behavior. Moreover, OC users experienced higher overall social and physical attraction. However, our results do not support the postulated roles for estradiol and progesterone on socio-sexual behavior in normally cycling women.

## Conclusions

Cycle-related changes in sleep, activity and physical discomfort do not appear to have much bearing on socio-sexual interest, nor do such behaviors differ with oral contraceptive use. However, attraction did appear to differ with OC usage. Findings from this study add to the current body of knowledge, by demonstrating that 1) a broader range of hormones is involved in female socio-sexual interest than is currently investigated, 2) women who utilize OCs report consistently more interest in social and sexual endeavors compared to their naturally cycling counterparts, and 3) socio-sexual interest in women using OCs may be modulated by different underlying hormonal patterns.

## List of Abbreviations

ANOVA: analysis of variance; CLIA: Clinical Laboratory Improvement Amendments; DHEAS: dehydroepiandrosterone sulfate; ELISA: enzyme-linked immunosorbent assay; OC: oral contraceptive; PASW: Predictive Analytic Software; SD: standard deviation; SPSS: Statistical Package for the Social Sciences; T1: early- to mid-follicular phase; T2: mid-cycle; T3: mid- to late-luteal phase.

## Competing interests

Co-author, DZ and ZRT Laboratory furnished the hormone assay kits and conducted all analysis at ZRT. All other authors report no conflict of interest.

## Authors' contributions

CG designed the study, wrote the protocol and methods section of the manuscript, recruited participants, and conducted the statistical analyses. HM recruited and collected data from participants, helped write the first draft of the manuscript, and managed the literature review. PG contributed to the study design and wrote the first draft of the manuscript. DZ approved the protocol, as well as contributed to the interpretation and discussion of findings. CM advised in design and protocol, added to the literature review, and wrote the final version of the manuscript. All authors edited the countless revisions and have approved the final manuscript.

## Pre-publication history

The pre-publication history for this paper can be accessed here:

http://www.biomedcentral.com/1472-6874/10/19/prepub
